# Preliminary Study of *CCR9* and *MAdCAM-1* Upregulation and Immune Imbalance in Canine Chronic Enteropathy: Findings Based on Histopathological Analysis

**DOI:** 10.3390/ani15121710

**Published:** 2025-06-10

**Authors:** Macarena Pino, Galia Ramirez, Caroll Beltrán, Eduard Martinez, Ismael Pereira, Jaime Villegas, Federico Cifuentes, Daniela Siel

**Affiliations:** 1 Escuela de Medicina Veterinaria, Facultad de Medicina y Ciencias de la Salud, Universidad Mayor, Santiago 8200010, Chile; macarena.pino@ug.uchile.cl (M.P.); ismael.pereira@umayor.cl (I.P.); 2 Central Veterinary Research Laboratory (LaCIV), Facultad de Ciencias Veterinarias y Pecuarias, Universidad de Chile, Santiago 8330015, Chile; galiaram@uchile.cl; 3 Laboratory of Immunogastroenterology, Biochemistry and Immunology Department, Faculty of Pharmacy, Universidad de Concepción, Concepción 4070386, Chile; cbeltranm@udec.cl; 4 Medicine Faculty, Universidad de Chile, Santiago 8330015, Chile; 5 Centro de Biomedicina, Universidad Mayor, Santiago 7500994, Chile; eduard.m.camacaro@gmail.com; 6 Escuela de Medicina Veterinaria, Facultad de Ciencias de la Vida, Universidad Andrés Bello, Santiago 8370035, Chile; jaime.villegas@unab.cl; 7 Escuela de Medicina Veterinaria, Facultad de Agronomía e Ingeniería Forestal, Facultad de Ciencias Biológicas y Facultad de Medicina, Pontificia Universidad Católica de Chile, Santiago 8331150, Chile; cifuentesff@gmail.com; 8 ESPA Diagnóstico Ltd.a., Santiago 7500961, Chile

**Keywords:** chronic inflammatory enteritis, canine, cytokines, chemokines, immune profile, MAdCAM-1, CCR9

## Abstract

Canine chronic enteropathy (CE) is a gastrointestinal disorder characterized by persistent or recurrent digestive symptoms lasting more than three weeks. It shares similarities with human inflammatory bowel disease but its immunopathogenesis remains poorly characterized in dogs. The aim of this study was to characterize the local and systemic immune profile of dogs with CE by assessing cytokine and chemokine expression in serum and intestinal tissue, as well as the mRNA expression of immune-related receptors such as integrins, chemokine receptors, and cytokines. Duodenal biopsies and blood samples were collected from five dogs diagnosed with a CE and five healthy controls. Serum concentrations of cytokines and chemokines were determined by multiplex ELISA, and mRNA expression in the intestinal mucosa was analyzed by quantitative PCR. Dogs with a CE showed increased expression of pro-inflammatory cytokines, including *TNF-α* and *IFN-γ*, and increased concentrations of chemokines such as CXCL10 and CCL2 in both serum and tissue samples. Increased mRNA expression of the chemokine receptor *CCR9* and the adhesion molecule *MAdCAM-1* were also observed in intestinal samples. These findings provide new insights into the immune response involved in CE and may aid the development of future diagnostic biomarkers and targeted therapies for canine chronic enteropathies.

## Simple Summary

Chronic enteropathy (CE) is a long-term digestive condition in dogs that can cause symptoms such as diarrhea, vomiting, and weight loss over a period of weeks or months. It is similar to a condition in humans called inflammatory bowel disease. However, the role of the immune system in this disease in dogs is not yet fully understood. In this study, we examined blood and gut samples from dogs with a CE, comparing them to samples from healthy dogs. We examined molecules that help the immune system send signals and manage inflammation. We found that dogs with CEs had higher concentrations of these molecules, both in their blood and in their intestines. We also observed changes in other immune molecules that guide immune cells to the gut. These findings improve our understanding of the immune system in dogs with CE and could lead to more effective diagnostic and treatment tools in the future.

## 1. Introduction

Chronic enteropathies (CEs) in dogs are a group of gastrointestinal disorders that persist for three or more weeks, fail to respond to symptomatic treatment, and are diagnosed when other digestive or extra-digestive diseases that cause similar symptoms have been excluded, with histopathological evidence of inflammation [1,2,3]. Historically referred to as inflammatory bowel disease (IBD) in analogy to the human condition, there is now increasing consensus to use the term CE to better reflect their distinct pathogenesis and classification [4].

Unlike the extensively studied human IBD, canine CEs remain incompletely characterized. CEs are currently classified based on clinical response to sequential treatment trials, resulting in categories such as food-responsive enteropathy (FRE), immunosuppressant-responsive enteropathy (IRE), and non-responsive enteropathy (NRE). Historically, antibiotic-responsive enteropathy (ARE) was included; however, recent studies have highlighted concerns regarding the long-term effects of antibiotics on the gut microbiota and the development of antimicrobial resistance. Consequently, there is a growing consensus to replace the ARE category with microbiota-related modulation-responsive enteropathy (MrMRE), emphasizing treatments aimed at restoring a healthy gut microbiota, such as prebiotics, probiotics, and fecal microbiota transplantation. Histopathological findings, including the affected gastrointestinal segment (e.g., enteritis, colitis, or enterocolitis) and the predominant type of cellular infiltrate (e.g., lymphocytic–plasmacytic, eosinophilic, neutrophilic, or granulomatous), continue to provide valuable information for characterizing the disease [5,6,7]. The most commonly reported histopathological form of CE in dogs is lymphocytic–plasmacytic enteritis (LPE), characterized by infiltration of lymphocytes and plasma cells within the lamina propria of the small intestine [8,9,10,11].

In human IBD, the predominant immune profiles of its two main forms—ulcerative colitis (UC) and Crohn’s disease (CD)—have been well described, with strong correlations established between immune patterns, histopathological features, and disease progression. This understanding has facilitated earlier diagnosis and the development of more targeted therapies. Conversely, in canine chronic enteropathy (CE), the limited understanding of the underlying immune mechanisms prolongs the diagnostic process, complicates treatment decisions, and affects the quality of life of both patients and their caregivers [12,13,14].

Deciphering the immunological landscape and its relationship to histopathological findings and clinical signs is essential. A deeper understanding of the immunopathogenesis of CE will support the development of novel tools to evaluate treatment efficacy, as well as the identification of new diagnostic markers and therapeutic targets to improve disease management in dogs [15].

The aim of this study was to characterize the predominant local and systemic immune profiles in dogs diagnosed with CE. To achieve this, we evaluated key immune parameters, including the quantification of cytokines and chemokines in both serum and intestinal tissue. In addition, we analyzed the mRNA expression of critical receptors such as integrins and chemokine receptors, as well as the mRNA expression of selected cytokines in the intestinal mucosa. These analyses provided new insights into the immune mechanisms underlying CE and may contribute to the identification of novel diagnostic markers and therapeutic targets.

## 2. Materials and Methods

### 2.1. Ethical Approval and Animal Selection

All procedures involving animals were approved by the Institutional Animal Care and Use Committee (CICUA) of the University of Chile, 19243-VET-UCH. Written informed consent was obtained from all dog owners prior to inclusion in the study.

A total of 10 client-owned dogs were enrolled and divided into two groups: dogs with a confirmed diagnosis of chronic enteropathy (CE, n = 5) and clinically healthy control dogs (n = 5). Animals were aged between 2 and 10 years, with no restriction on sex or breed. CE was diagnosed based on chronic gastrointestinal signs lasting ≥3 weeks, histopathological evidence of intestinal mucosal inflammation, and the exclusion of other potential underlying causes, including exocrine pancreatic insufficiency, parasitic diseases, and hypoadrenocorticism [1]. The dogs had all been put on an exclusion diet, either based on hydrolyzed or novel protein, but this treatment had a partial or poor effect on them. As this was a pilot study, we did not monitor the patients to ascertain whether they subsequently responded to alternative treatments, such as immunosuppressants, fecal microbiota transplantation, or cholestyramine, or whether they were ultimately categorized as non-responsive cases.

Control dogs were client-owned animals with no history of gastrointestinal or systemic disease in the previous 12 months. All control dogs underwent a thorough physical examination, complete blood count, serum biochemistry, and fecal examination and were included only if no abnormalities were detected. These animals had not received any immunosuppressive, antimicrobial, or anti-inflammatory medications for at least three months prior to inclusion.

### 2.2. Sample Collection

All dogs underwent physical examination, blood sampling, and gastroduodenoscopy under general anesthesia. Blood samples (3–5 mL) were collected from the cephalic vein into tubes with and without EDTA for hematology, biochemistry, and cytokine/chemokine analysis.

Endoscopic biopsies were performed under standardized anesthetic protocols using a flexible videoendoscope and biopsy forceps. Eight duodenal mucosal biopsies were collected per animal: five were preserved in formalin for histopathological examination and three were stored in RNA later at −80 °C for subsequent molecular analysis.

### 2.3. Quantification of Serum Cytokines and Chemokines

Serum concentrations of cytokines and chemokines were quantified using a multiplex ELISA platform. Plasma samples were shipped on dry ice to EveTechnologies (Rockville, MA, USA) for analysis using the Canine Cytokine/Chemokine 13-Plex Discovery Assay^®^ (Newmarket, ON, Canada). The selected markers evaluated in this study included IL-8/CXCL8, CXCL10, CCL2, IL-18, TNF-α, and IFN-γ.

### 2.4. Gene Expression Analysis in Intestinal Tissue

The total RNA was extracted from intestinal biopsies using the RNeasy Mini Kit (Qiagen, Hilden, Germany), followed by DNase I treatment. The amount and purity of RNA were assessed spectrophotometrically. Complementary DNA (cDNA) synthesis was performed using the ImProm-II™ Reverse Transcription System (Promega, Madison, WI, USA).

Quantitative PCR (qPCR) was performed using the Brilliant II SYBR^®^ Green QPCR Master Mix Kit (Agilent Technologies, Santa Clara, CA, USA) in a StepOnePlus™ Real-Time PCR System (Applied Biosystems, San Francisco, CA, USA). Gene expression was analyzed for target cytokines, chemokines, and their receptors, including key molecules such as integrins and CCR9. Expression values were normalized to reference genes and calculated using the 2^−ΔΔCt^ method.

### 2.5. Statistical Analysis

Data were analyzed using GraphPad Prism 8.0. The Shapiro–Wilk test was used to assess the normality of the data. Comparisons between groups were made using unpaired *t*-tests for normally distributed data or Mann–Whitney U tests for non-parametric data. A *p*-value < 0.05 was considered statistically significant.

## 3. Results

### 3.1. Study Population

**Figure 1:**
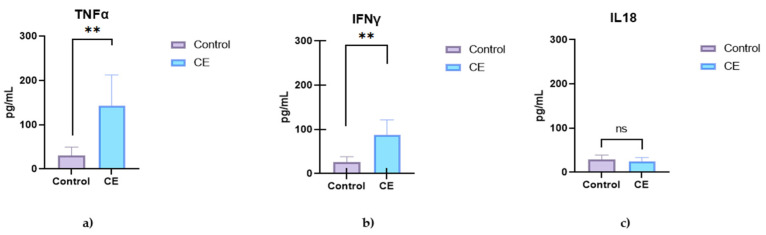
Serum cytokine concentrations in healthy control dogs (n = 5) and dogs with chronic enteropathy (CE; n = 5). The graphs show the concentrations of (**a**) interferon-γ (IFN-γ), (**b**) tumor necrosis factor-α (TNF-α), and (**c**) interleukin-18 (IL-18). Data are presented as single values with a median. Asterisks (**) indicate statistically significant differences (*p* < 0.05); ns = not significant.

A total of 10 dogs from Santiago, Chile, were included in this study. It was conducted at the Hospital Veterinario MEDIVET and the Centro de Diagnóstico SEDIVET. Five dogs were assigned to the control group and five to the CE group. All dogs in the experimental group were diagnosed according to the previously mentioned criteria, including histopathological analysis of duodenal biopsies. Macroscopic examination of the duodenum in dogs with chronic enteropathy (CE) revealed consistent inflammatory changes, including mucosal edema, erythema, friability, and visible lacteal dilations in one of the affected dogs, suggestive of intestinal lymphangiectasia. These lesions were markedly different from those in healthy control dogs, whose duodenal mucosa appeared mildly irregular with subtle discoloration but lacked overt inflammatory features. Histopathological evaluation confirmed the presence of lymphoplasmacytic infiltrates and stromal fibrosis in the lamina propria of all CE dogs, consistent with a diagnosis of lymphoplasmacytic enteritis. In contrast, biopsies from healthy dogs showed normal histological architecture without evidence of inflammation or fibrosis (Figure 1a).

Detailed information on breed, sex, and disease severity scores is given in Table A2. A summary of hematological and biochemical analyses for both groups is given in Table A3.

### 3.2. Serum Cytokine and Chemokine Concentrations

**Figure 2:**
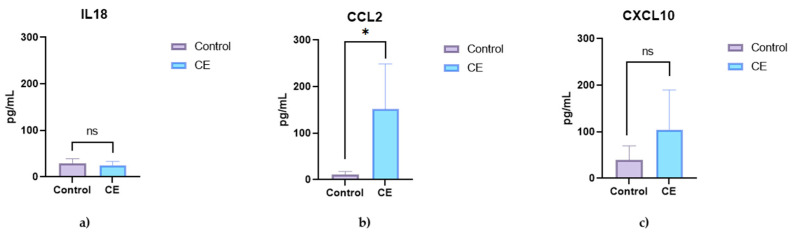
Serum chemokine concentrations in healthy control dogs (n = 5) and dogs with chronic enteropathy (CE; n = 5). The graphs show the concentrations of (**a**) IL-8 (CXCL8), (**b**) CXCL10, and (**c**) CCL2. Data are presented as single values with a median. Asterisks (*) indicate statistically significant differences (*p* < 0.05); ns = not significant.

Serum analysis revealed significantly elevated concentrations of pro-inflammatory cytokines in dogs with chronic enteropathy (CE) compared to healthy controls (Figure 1). Interferon-gamma (IFN-γ) and tumor necrosis factor-alpha (TNF-α) were significantly increased in the CE group (*p* = 0.0051 and *p* = 0.0082, respectively), whereas interleukin-18 (IL-18) showed a non-significant increase (*p* = 0.4836) (Figure 1). Among chemokines, IL-8 (CXCL8) and CCL2 were significantly elevated in CE dogs (*p* = 0.0004 and *p* = 0.0122, respectively), whereas CXCL10 showed a non-significant trend toward elevation (*p* = 0.1439) (Figure 2).

### 3.3. Intestinal Gene Expression of Immune Mediators

**Figure 3:**
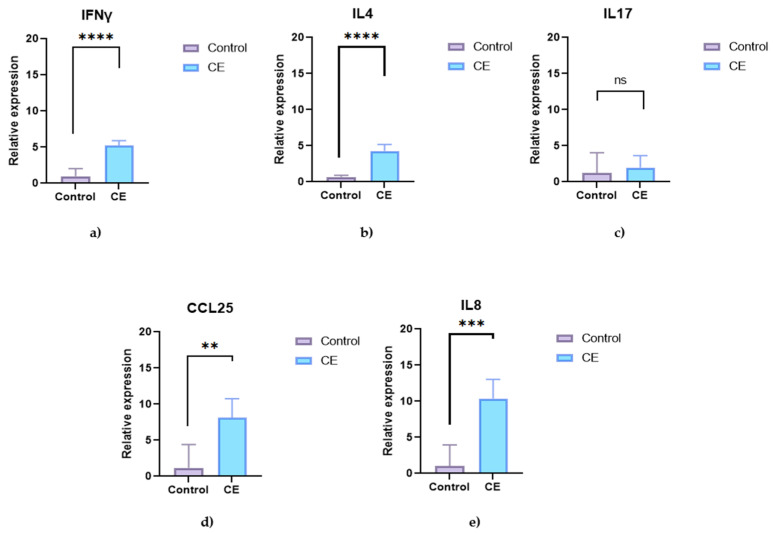
Relative mRNA expression in the duodenal mucosa of healthy control dogs (n = 5) and dogs with chronic enteropathy (CE; n = 5). The graphs show the expression of (**a**) *interferon-γ (IFN-γ)*, (**b**) *interleukin-4 (IL-4)*, (**c**) *interleukin-17 (IL-17)*, (**d**) *CCL25,* and (**e**) *CXCL8 (IL-8).* Data are presented as single values with a median. Asterisks (*) indicate statistically significant differences (** *p* < 0.01; *** *p* < 0.001; **** *p* < 0.0001; ns = not significant (*p* ≥ 0.05)).

Quantitative PCR analysis of duodenal biopsies showed a significant upregulation of *IFN-γ* and *IL-4* mRNA in dogs with CE compared to controls (*p* < 0.0001 for both). *IL-17* expression was also increased but did not reach statistical significance (*p* = 0.5243) (Figure 3). The chemokines *CCL25* and *CXCL8 (IL-8)* were both significantly increased in the intestinal tissue of affected dogs (*p* = 0.0016 and *p* = 0.0002, respectively) (Figure 3).

#### 3.3.1. Transcription Factors in Intestinal Tissue

**Figure 4:**
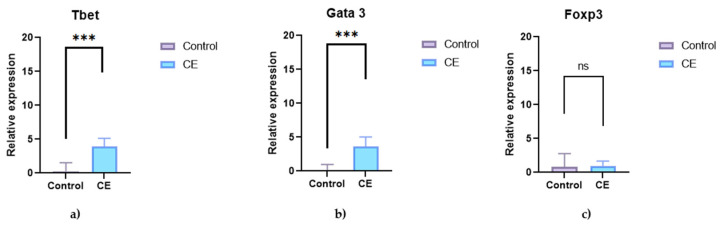
Relative mRNA expression of transcription factors in the duodenal mucosa of healthy control dogs (n = 5) and dogs with chronic enteropathy (CE; n = 5). The graphs show expression of (**a**) *T-bet*, (**b**) *GATA3*, and (**c**) *Foxp3*. Data are presented as single values with a median. Asterisks (***) indicate statistically significant differences (*p* < 0.05); ns = not significant.

The analysis of transcription factors showed significantly higher expression of *T-bet* and *GATA3* in the CE group (*p* = 0.0004 and *p* = 0.0003, respectively). *Foxp3* expression was reduced in dogs with CE, but the difference was not statistically significant (*p* = 0.8591) (Figure 4).

#### 3.3.2. Expression of *CCR9*, *CCR2*, *CCR3*, and *MAdCAM-1* in Duodenal Mucosa

**Figure 5:**
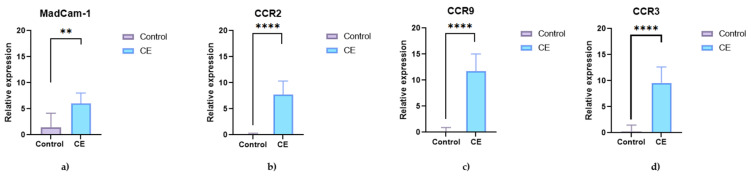
Relative mRNA expression of adhesion and chemokine receptors in the duodenal mucosa of healthy control dogs (n = 5) and dogs with chronic enteropathy (CE; n = 5). The graphs show expression of (**a**) *MAdCAM-1*, (**b**) *CCR2*, (**c**) *CCR9*, and (**d**) *CCR3*. Data are presented as single values with a median. Asterisks (*) indicate statistically significant differences (** *p* < 0.01; **** *p* < 0.0001).

A significant increase in mRNA expression of *CCR9* was observed in the intestinal mucosa of dogs with CE compared to controls (*p* < 0.0001), along with increased expression of *CCR2* and *CCR3* (*p* < 0.0001 for both). In addition, mucosal expression of *MAdCAM-1* was significantly higher in dogs with CE (*p* = 0.0056), suggesting increased lymphocyte trafficking and mucosal immune activation in these animals (Figure 5).

## 4. Discussion

Canine chronic enteropathy (CE) shares histopathological features with human inflammatory bowel disease (IBD), but the immunological mechanisms involved remain poorly defined in the canine species. The dynamic interplay between the innate and adaptive branches of the immune system plays a central role in the pathogenesis of CE. This complex immunological crosstalk contributes to the initiation and perpetuation of intestinal inflammation, involving both early, nonspecific responses and highly regulated antigen-specific mechanisms [16]. Understanding these immune pathways not only provides insight into disease progression but also opens new opportunities for the identification of reliable diagnostic and prognostic biomarkers, as well as for the development of targeted immunomodulatory therapies.

In this study, we characterized the local and systemic immune response in dogs with CE by assessing cytokine and chemokine concentrations in serum and intestinal tissue and the expression of transcription factors and key receptors such as *CCR9* and *MAdCAM-1*. A key finding was the significant upregulation of *CCR9* and its ligand *CCL25*, together with the adhesion molecule *MAdCAM-1*, in the duodenal mucosa of dogs with CE. These molecules play a central role in lymphocyte homing to the gut and have been described as key elements in the pathogenesis and treatment of human IBD [17,18]. Additionally, the increased expression of *CCR2* and *CCR3* supports the hypothesis of enhanced immune cell recruitment to inflamed intestinal tissue in CE.

In dogs with CE, a loss of immune tolerance at the intestinal mucosa has been documented. This is characterized by dysfunction in pattern recognition receptors, such as TLR4 and TLR5, as well as local overexpression of inflammatory cytokines, including TNF-α, IL-8, and IFN-γ. Histologically, this immune dysregulation is reflected by lymphoplasmacytic infiltration and an increased number of CD4^+^ T cells, as well as a loss of mucosal architecture [13,14,15,16,17,18,19].

Unlike the clear Th1 or Th2 polarization observed in certain human IBD subtypes, canine IBD does not consistently exhibit a dominant cytokine profile. Instead, the immune response appears to be heterogeneous, as shown by previous meta-analyses and gene expression studies [16,19,20]. A study by Ohta et al. (2014) [21] found no significant differences in IL-17A, IFN-γ, IL-4, or IL-10 expression at either the mRNA or protein level in the duodenal mucosa of dogs with IBD compared to controls. These findings suggest that typical T cell cytokine-driven inflammation may not be central to the pathogenesis of lymphoplasmacytic enteritis in dogs [21]. Similarly, Schmitz et al. (2012) [22] reported a significant decrease in *IL-17A* mRNA expression in the duodenal mucosa of dogs with IBD. No significant changes were observed for IL-22, IFN-γ, IL-10, or TGF-β. These results provide further support for the idea that canine IBD lacks a consistent Th17 or Th1/Th2 cytokine profile. They also highlight the complexity of the mucosal immune response and suggest that canine IBD may differ from human IBD in terms of immunopathogenesis [22]. Consistent with this, Tamura et al. (2014) [23] examined the expression of cytokine mRNA in the colonic mucosa of dogs with lymphocytic–plasmacytic colitis and found no significant differences in Th1 (*IFN-γ*), Th2 (*IL-4*), Th17 (*IL-17*), or regulatory (*IL-10*) cytokines compared to healthy controls. Although *IL-23p19* was elevated, most cytokines, including several key pro-inflammatory markers, showed no differential expression. This further highlights the absence of a dominant T helper cytokine pattern in this type of canine CE, supporting the notion of a unique immunopathogenic process in dogs [23].

In this study, cytokine analysis revealed significantly higher concentrations of IFN-γ and TNF-α in dogs with CE, consistent with previous findings suggesting a Th1-skewed response in canine CE and human Crohn’s disease (CD) [24,25]. In line with these results, increased serum IL-8 (CXCL8) and CCL2 (MCP-1) concentrations were also observed, highlighting systemic immune activation and their likely role in leukocyte recruitment [26,27]. These findings are consistent with those reported by Kaga et al. (2024), who found elevated serum IL-8 and MCP-1 in dogs with CE [25]. Given the complexity of the immune response in CE, future studies should also consider the involvement of additional mediators such as interleukin-1 (IL-1) and IgA. In this regard, Maeda et al. (2012) previously demonstrated a mucosal imbalance between IL-1β and its receptor antagonist in dogs with CE, which likely contributes to sustained intestinal inflammation [28]. Furthermore, in a subsequent study, Maeda et al. (2013) identified reduced concentrations of immunoglobulin A (IgA) in feces, duodenal mucosa, and peripheral blood mononuclear cells of dogs with CE, supporting the hypothesis of impaired mucosal immunity in these patients [29].

At the intestinal mucosal level, our study showed increased expression of *IFN-γ* and *IL-4* mRNA, accompanied by upregulation of the transcription factors *T-bet* and *GATA3,* suggesting a mixed Th1/Th2 immune profile. This aligns with prior studies indicating that dogs with CE may exhibit variable Th polarization depending on disease phase and tissue localization [9]. In contrast, we found no significant differences in the expression of IL-17 or FoxP3, limiting conclusions regarding the role of Th17 and regulatory T cells, although the trend toward higher IL-17 expression merits further investigation, especially given its relevance in human IBD pathogenesis [30].

At the intestinal mucosal level, our study showed increased expression of *IFN-γ* and *IL-4* mRNA, accompanied by upregulation of the transcription factors *T-bet* and *GATA3*, suggesting a mixed Th1/Th2 immune profile. This aligns with prior studies indicating that dogs with CE may exhibit variable Th polarization depending on disease phase and tissue localization [9]. In contrast, we found no significant differences in the expression of *IL-17* or *FoxP3*, limiting conclusions regarding the role of Th17 and regulatory T cells, although the trend toward higher IL-17 expression merits further investigation, especially given its relevance in human IBD pathogenesis [28]. Our findings are also consistent with those of Konstantinidis et al. (2021), who reported increased expression of *IL-1β*, *IL-23p19,* and *CCL28* in colonic mucosa from dogs with large intestinal IBD [31], reinforcing the role of innate immune mediators and mucosal inflammation in CE.

In future studies, our findings could be complemented by the phenotypic characterization of peripheral and intestinal lymphocyte populations. A recent study has shown increased circulating activated CD4⁺CD25⁺ T helper and regulatory T cells (CD4⁺CD25⁺FoxP3⁺), as well as a shift toward intestinal CD8⁺ T cell dominance and increased IFN-γ production in CE dogs. These alterations parallel immune signatures in human IBD and suggest potential for translational research and development of immune-based biomarkers [32].

A key finding of this study was the significant upregulation of *CCR9* and its ligand *CCL25*, together with the adhesion molecule *MAdCAM-1*, in the duodenal mucosa of dogs with CIE. These molecules play a central role in the homing of lymphocytes to the gut and have been described as key elements in the pathogenesis and treatment of IBD in humans [17,18]. The increased expression of *CCR2* and *CCR3* observed in this study further supports the hypothesis of increased recruitment and trafficking of immune cells to inflamed intestinal tissue in canine CE.

Collectively, these findings provide novel insights into the immunopathogenesis of canine CE and suggest that affected dogs may exhibit immune signatures that mirror those observed in human IBD [24,25,26,27]. In particular, the identification of increased expression of *CCR9* and *MAdCAM-1* supports the existence of conserved gut-homing mechanisms across species and highlights their potential as translational targets for both diagnostic development and therapeutic intervention.

Nonetheless, certain limitations of this study should be acknowledged. The relatively small sample size may limit the generalizability of our findings, and disease severity was assessed exclusively through histopathological evaluation. Future studies would benefit from the inclusion of standardized clinical scoring systems (e.g., CCECAI or CIBDAI) at diagnosis, alongside longitudinal tracking of treatment response. This would enable the classification of dogs by CE subtype (e.g., food-responsive, antibiotic-responsive, immunosuppressive-responsive), allowing more precise correlation between molecular markers and clinical phenotypes.

Further research is warranted to explore the prognostic potential of the immunological markers identified in this study, including their relationship with long-term therapeutic outcomes. In addition, deeper investigation into the use and variability of clinical scoring indices may uncover their utility as predictors of disease progression, ultimately supporting a more personalized and timely therapeutic approach in veterinary medicine.

## 5. Conclusions

This study provides novel evidence of systemic and intestinal immune dysregulation in dogs with chronic inflammatory enteritis (CE). Dogs with CE exhibited elevated serum concentrations of pro-inflammatory cytokines and chemokines, as well as increased expression of Th1- and Th2-associated markers in the intestinal mucosa. Significant upregulation of *CCR9*, *CCL25*, and *MAdCAM-1* suggests increased lymphocyte trafficking to the gut, similar to mechanisms described in human inflammatory bowel disease. These findings contribute to the understanding of the immunopathogenesis of CE and highlight potential molecular targets for future diagnostic and therapeutic strategies in canine chronic enteropathies.

## Figures and Tables

**Figure A1:**
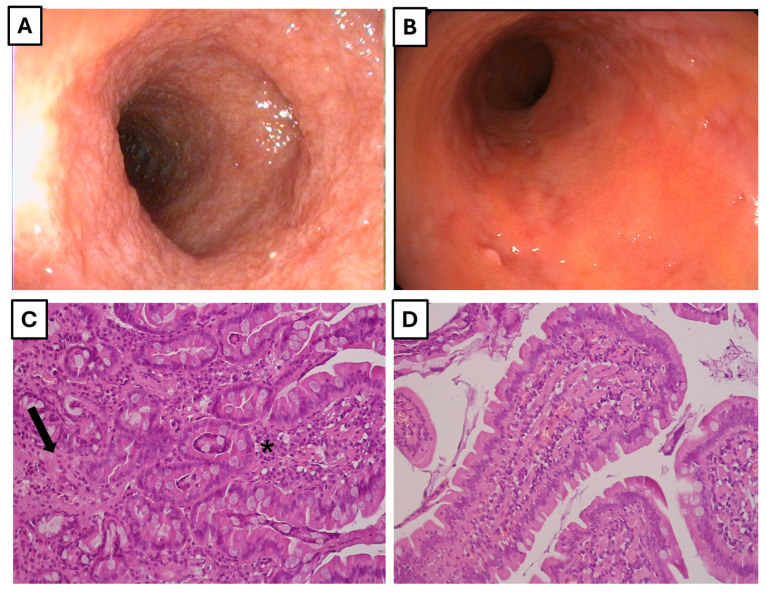
Macroscopic and microscopic evaluation of the duodenum in dogs. (**A**) Representative duodenal image from a dog with chronic enteropathy (CE), showing macroscopic lesions associated with inflammation. (**B**) Duodenum from a healthy control animal, showing mildly irregular mucosa and subtle discoloration. (**C**) Duodenal biopsy from a CE dog, exhibiting lymphoplasmacytic infiltrate (asterisk) and stromal fibrosis (arrow) in the lamina propria. (**D**) Duodenal biopsy from a healthy dog, with no histological abnormalities. All dogs in the CE group were diagnosed with lymphoplasmacytic enteritis based on histopathological analysis.

## Data Availability

The original contributions presented in this study are included in the article. Further inquiries can be directed to the corresponding author.

## Appendix A

**Table A1:** Analyzed nucleotide sequencing of molecules in samples of the small intestine according to the T helper response.

T Cell Response	mRNA Analyzed	Sequencing Primers
**Th1**	Tbet F	AATCAGCACCAGACGGAGAT
	Tbet R	GTCCACGAACATCCGGTAAT
	IFN-γ DogF1	GCGCAAGGCGATAAATGAAC
	IFN-γ DogR1	CTGACTCCTTTTCCGCTTCC
**Th2**	Gata 3 F	TACGTCCCCGAATACAGCTC
	Gata 3 R	ACTCCCTGCCTTCTGTGCT
	Gata 3 F	CGAAGGCTGTCGGCAGCAAGAA
	Gata 3 R	ACGGGGTCTCCGTTGGCATT
	IL-4 DogF1	TCACCAGCACCTTTGTCCAC
	IL-4 DogR1	CGCTTGTGTTCTTTGGAGCA
	IL-17 DogF	GGAATCTGCACCGCAATGAGGAC
	IL-17 DogR	CGCAGAACCAGGATCTCTTGCTGG
**Treg**	Foxp3 F	CAAATGGTGTCTGCAAGTGG
	Foxp3 R	GTGCTCTGCCCTTCTCATCT
	IL-10 DogF1	CGGGAGGGTGAAGACTTTCT
	IL-10 DogR1	GGCATCACCTCCTCCAAGTA

**Table A2:** Analyzed nucleotide sequencing of molecules in samples of the small intestine implicated in “homing”.

Molecule	Sequence
α4β7 F receptor MAdCAM-1 F	CCCTACCAGCTCAGCAGAGGACA
α4β7 F receptor MAdCAM-1 R	ACCCGGGCTACACCCTCGTC
CCR9 F	CACTTCCTCCCACCCTTGTA
CCR9 R	TGGTCTTGACTCTGGTGCAG
CCR2 F	CGTGAAGCTCATTTTCGTGA
CCR2 R	GACTCCTGGAAGGTGCTCAG
CCR3 F	TCTTCCACGAGTCCCAAA AG
CCR3 R	GAGCAGAGGCAGAGCAAGAC

**Table A3:** Mucosal biopsies from dogs with CE.

Animal Number	Breed	Age	Genre	Histopathological Severity
1	German shepherd	3 years	Male	Severe
2	Mixed	6 years	Female	Severe
3	French bulldog	3 years	Male	Moderate
4	Labrador	6 years	Female	Moderate
5	Mixed	5 years	Male	No data

**Table A4:** Mucosal biopsies from healthy dogs.

Animal Number	Breed	Age	Genre
1	Mixed	7 years	Female
2	Mixed	8 years	Female
3	Golden Retriever	7 years	Male
4	Mixed	5 years	Male
5	Mixed	6 years	Male
